# Demoralization of Inferior Work

**Published:** 1911-06-15

**Authors:** Orison Swett Marden


### DEMORALIZATION OF INFERIOR WORK.

BY ORISON SWETT MARDEN.

Nothing kills ambition or lowers the life standard quicker than familiarity with inferiority—that which is cheap, the "Cheap John" method of doing things. We unconsciously become like that with which we are habitually associated. It becomes part of us, and the habit of doing things in an inferior, slovenly way weaves its fatal defects into the very texture of the character.

We are so constituted that the quality which we put into our lifework affects everything else in our lives, and tends to bring our whole conduct to the same level. The whole person takes on the characteristics of one's usual way of doing things.

The habit of precision and accuracy affects the entire mentality, improves the whole character.

On the contrary, doing things in a loose jointed, slipshod, careless manner deteriorates the whole mentality, demoralizes the entire mental processes, and brings down the whole life.

Every half done or slovenly job that goes out of your hands leaves its trace of demoralization behind, takes a bit from your self-respect. After slighting your work, after doing a poor job, you are not quite the same man you were before. You are not so likely to try to keep up the quality of your work, not so likely to regard your word as sacred as before. You incapacitate yourself from doing your best in proportion to the number of times you allow yourself to do inferior, slipshod work.

The mental and moral effect of half-doing, or carelessly doing things; its power to drag down, to demoralize, can hardly be estimated, because the processes are so gradual, so subtle. No one can respect himself who habitually botches his work, and when self-respect drops, confidence goes with it; and when confidence and self-respect have gone, excellence is impossible.

It is astonishing how completely a slovenly habit will gradually, insidiously fasten itself upon the individual and so change his whole mental attitude as to thwart absolutely his life-purpose, even when he may think he is doing his best to carry it out.

One's ambition and ideals need constant watching and cultivation, in order to keep the standards up. Many people are so constituted that their ambition deteriorates and their ideals drop when they are alone, or with careless, indifferent people. They require the constant assistance, suggestion, prodding, or example, of others to keep them up to standard.—*The Busy Man's.*

